# COVID-19 and the Lebanese Crisis: Will the Phoenix Manage to Rise Once Again?

**DOI:** 10.1017/dmp.2020.416

**Published:** 2020-10-26

**Authors:** Abdul Rahman Bizri, Walid Alam, Nazih A. Bizri, Umayya Musharrafieh

**Affiliations:** 1 American University of Beirut Medical Center, Department of Internal Medicine, Division of Infectious Diseases, Director Quality Program, COVID-19 task force Lebanese Ministry of Public Health, National Expert Group on Transmissible Diseases Lebanese Ministry of Public Health; 2 American University of Beirut Medical Center, Department of Internal Medicine; 3 University of Balamand, Faculty of Medicine; 4 American University of Beirut Medical Center, Department of Family Medicine, Vice Chair, Professor of Medicine, Director of COVID-19 Clinic

A series of unfortunate events has recently rocked Lebanon. The October 17, 2019 uprising took place against the political ruling class and the management of the country’s affairs.^1^ The political stability index in 2019 was –1.64 with the score ranging from –2.5 (weak) to 2.5 (strong). Civil unrest arose due to monetary collapse, state bankruptcy, devaluation of the local currency and deep recession.^2^ The resulting financial and social instabilities have forced a great number of well-trained and highly skilled physicians and health-care workers to leave the country seeking better employment opportunities.^3,4^. Major medical centers in the country have uncollected debt mainly from the government accounting for hundreds of millions of US dollars that are likely to be written off due to the financial crisis.^5^

The coronavirus disease 2019 (COVID-19) pandemic could not have hit Lebanon at a worse time, adding insult to injury when the health-care system was unprepared, and resource depleted.^6^ To prevent a massive first wave of severe acute respiratory syndrome coronavirus 2 (SARS-CoV-2) cases, the government employed a complete shutdown of the country.^7^ This led to further economic depression and increase in joblessness and poverty. Reversal of measures were gradually implemented in an attempt to rescue a flailing economy, as schools and places of worship were reopened, and all shops were allowed to re-operate at up to 50% capacity. Curfew hours were reduced, and the public was encouraged to maintain social distancing and wear masks. The outcome was almost catastrophic as COVID-19 numbers soared to 224 cases/d compared with a median of roughly 40 cases/d, burdening the health-care system even further.^8^ To make matters worse, an unexpected devastation took place, where an enormous explosion at the Port of Beirut on August 4, 2020, happened, resulting in more than 5000 injuries, around 200 deaths, and 300,000 people made homeless. Several university medical centers and tertiary hospitals located within 1.5 km radius were severely damaged and were put out of business for weeks and possibly months. Other major medical centers outside this radius were overwhelmed with casualties despite being partially damaged.^9^ Intensive care units, already saturated with critical COVID-19 patients, had to cope with seriously wounded individuals. What they experienced was close to a logistical nightmare, necessitating the re-allocation of patients among various wards and critical care units.

Most of the medical supplies imported to the country were stored at the Port of Beirut facilities. Some stocks were buried under rubble, while others destroyed. The World Health Organization (WHO) estimates 17 containers with essential medical supplies destroyed, 500 hospital bed equivalents lost, 3 hospitals rendered nonfunctional, and 3 hospitals substantially damaged.^10^ The blast was caused by an estimated amount of 2750 tons of highly explosive ammonium nitrate poorly kept under suboptimal conditions.^11^ The potential environmental consequences and their health effects are under evaluation and assessment by a special taskforce formed by the minister of health on August 7, 2020. The political repercussions of the massive explosion were tremendous. People went down to the streets in protest leading to violent confrontations with the armed forces. This resulted in large numbers of casualties and further loss of life on both sides. The humanitarian aftermath was even graver, where thousands of families became homeless; many had close relatives and friends missing, numerous businesses were destroyed, and some were unable to procure food or obtain their daily medications.

Both the political and humanitarian consequences have yielded additional pressure on an already fragile, overburdened, and resource depleted health-care system. Two weeks after the explosion, the Lebanese Ministry of Public Health announced 3241 new confirmed COVID-19 cases, representing almost one-third of all cases in Lebanon since the start of the pandemic on February 21, 2020. On Thursday August 20, a new record number of confirmed daily cases reaching 605 was announced.^12^ This prompted the government to implement a 2-wk national partial lockdown starting August 21, with a curfew in effect from 6 pm to 6 am.^13^ The effectiveness of a partial lockdown at this stage of the pandemic is debatable. Data from Lombardy, Veneto, and Emilia-Romagna, the 3 most affected regions of Italy, showed that less rigid lockdown led to an insufficient decrease in mobility to reverse an outbreak, such as COVID-19. Tighter restrictions led to a greater decrease in mobility, enough to bring down transmission promptly below the level needed to sustain the epidemic.^14^

Several questions were raised. Are the restrictions imposed by the lockdown enough to halt or slow the epidemic? Is the duration of 2 wk appropriate and satisfactory? Can a full lockdown be implemented given the current state of the economy? Many oppose the lockdown due to their need to work and earn their living. Others believe that we should let this pandemic run its course as initially illustrated by Sweden when the government refused to implement any lockdown measures and aimed to attain herd immunity recording the most coronavirus deaths per capita in Europe in a seven day average between 25 May and 2 June with a mortality rate reaching 5.29 deaths per million inhabitants a day.^15^

According to Nassim N. Taleb, the author of the best-selling book *Antifragile*,^16^ Lebanon has the potential for being antifragile. This means that the country is capable of rejuvenating to a better version after experiencing disorder or destruction, owing to the Lebanese culture and its people’s resilience in the face of adversity.^17^ Lebanon is usually referred to as the phoenix, a mythological bird that cyclically regenerates or is otherwise born again from ashes.^18^ The country’s long history is full of major crises that have threatened its sheer existence, yet the Lebanese have managed to rebound and rebuild their country and economy in an improved and more suitable form.^19,20^ Pandemics have often resulted in a series of political and economic ramifications as well as health consequences.^21–23^ Lebanon is not be the only nation hit by the COVID-19 pandemic while facing difficulties and unfavorable conditions. The Lebanese situation should be monitored and evaluated to help understand how to revive countries witnessing similar conditions during a major pandemic. However, what the health-care system in Lebanon is experiencing may be beyond the ability of the phoenix to recover.

## References

[1] Transparency international. The Lebanese Transparency Association. https://www.transparency.org/en/countries/lebanon#. Accessed December 3, 2020.

[2] Bidawi H . Large devaluation and inequality: the case of Lebanon (Note). BSI Economics. Published May 5, 2020. http://www.bsi-economics.org/1130-large-develuation-inquality-case-of-lebanon. Accessed December 3, 2020.

[3] *The Arab Weekly*. Lebanon’s turmoil sparks exodus of professionals, especially bi-nationals. Published July 21, 2020. https://thearabweekly.com/lebanons-turmoil-sparks-exodus-professionals-especially-bi-nationals. Accessed December 3, 2020.

[4] *Arabian Business*. How desperation is fueling a new exodus from crisis-hit Lebanon. Published October 10, 2020. https://www.arabianbusiness.com/culture-society/452962-how-desperation-is-fuelling-the-new-exodus-from-lebanon. Accessed December 3, 2020.

[5] Lebanon: hospital crisis endangering health. https://www.hrw.org/news/2019/12/10/lebanon-hospital-crisis-endangering-health. Accessed August 2020.

[6] Abdul Rahman Bizri , Hussein H. Khachfe , Mohamad Y. Fares & Umayya Musharrafieh (2020). COVID-19 pandemic: an insult over injury for Lebanon. J Community Health. 2020;1-7. doi: 10.1007/s10900-020-00884-y PMC7358300 32661861

[7] Coronavirus: Lebanon begins ‘total’ shutdown as cases increase. https://www.bbc.com/news/world-middle-east-52637725. Accessed August 2020.

[8] COVID-19 response – Lebanon bi-monthly situation report (29 May 2020). https://reliefweb.int/report/lebanon/covid-19-response-lebanon-bi-monthly-situation-report-29-may-2020. Accessed November 24, 2020.

[9] United Nations Office for the Coordination of Humanitarian Affairs (OCHA). Lebanon: Beirut port explosions. Situation report no. 1 as of 5 August 2020 https://reliefweb.int/sites/reliefweb.int/files/resources/Beirut%20Port%20SitRep%20No.%201.pdf. Accessed August 2020.

[10] World Health Organization (WHO). Lebanon explosion update for partners: August 18, 2020 https://www.who.int/emergencies/who-lebanon-partners-update-18august2020.pdf. Accessed August 2020.

[11] BBC News. Beirut explosion: what we know so far. https://www.bbc.com/news/world-middle-east-53668493. Accessed August 2020.

[12] Johns Hopkins University of Medicine, Coronavirus Resource Center. https://coronavirus.jhu.edu/region/lebanon. Accessed August 2020.

[13] BBC News. Coronavirus: Lebanon to impose lockdown as cases spike after explosion. https://www.bbc.com/news/world-middle-east-53833273. Accessed August 2020.

[14] Vinceti M , Filippini T , Rothman KJ , et al. Lockdown timing and efficacy in controlling COVID-19 using mobile phone tracking. EclinicalMedicine. 2020;25:100457. doi: 10.1016/j.eclinm.2020.100457 32838234 PMC7355328

[15] Habib H . Has Sweden’s controversial covid-19 strategy been successful? BMJ 2020;369:m2376. 32532807 10.1136/bmj.m2376

[16] Taleb NN . Antifragile: Things That Gain from Disorder. New York: Random House Trade Paperbacks; November 27, 2012.

[17] Taleb NN . Lebanon: from Ponzi to Antifragility. Medium. https://medium.com/@nntaleb/lebanon-from-ponzi-to-antifragility-c9867eb71998. Accessed December 3, 2020.

[18] Petri A . This ancient middle eastern country is a history buff’s dream, Lebanon is one of the world’s oldest countries. *National Geographic* https://www.nationalgeographic.com/travel/destinations/asia/lebanon/photos-beirut-history. Accessed December 3, 2020.

[19] Voa news. Beirut bounces back from civil war. https://www.voanews.com/world-news/middle-east-dont-use/beirut-bounces-back-civil-war. Accessed December 3, 2020.

[20] Centre for Public Impact. Backing Beirut to bounce back. https://www.centreforpublicimpact.org/backing-beirut-bounce-back/. Accessed December 3, 2020.

[21] Smith KM , Machalaba CC , Seifman R , et al Infectious disease and economics: the case for considering multi-sectoral impacts. One Health 2019;7:100080. doi: 10.1016/j.onehlt.2018.100080 30671528 PMC6330263

[22] Tisdell CA . Economic, social and political issues raised by the COVID-19 pandemic. Econ Anal Policy 2020;68:17–28. doi: 10.1016/j.eap.2020.08.002 32843816 PMC7440080

[23] Madhav N , Oppenheim B , Gallivan M , et al Pandemics: risks, impacts, and mitigation In: Jamison DT , Gelband H , Horton S , et al, eds. Disease Control Priorities: Improving Health and Reducing Poverty. 3rd ed. The International Bank for Reconstruction and Development/The World Bank; 2017 https://www.ncbi.nlm.nih.gov/books/NBK525302/. doi: 10.1596/978-1-4648-0527-1_ch17. Accessed December 3, 2020.

